# Findings From a National Survey of Older US Adults on Patient Willingness to Use Telehealth Services: Cross-Sectional Survey

**DOI:** 10.2196/50205

**Published:** 2024-05-23

**Authors:** Olufeyisayo O Odebunmi, Tamera D Hughes, Austin R Waters, Benjamin Y Urick, Caroline Herron, Mary Wangen, Catherine Rohweder, Renée M Ferrari, Macary W Marciniak, Stephanie B Wheeler, Alison T Brenner, Parth D Shah

**Affiliations:** 1 Department of Health Policy and Management University of North Carolina Chapel Hill, NC United States; 2 Lineberger Comprehensive Cancer Center University of North Carolina Chapel Hill, NC United States; 3 Eshelman School of Pharmacy University of North Carolina Chapel Hill, NC United States; 4 Prime Therapeutics Eagan, MN United States; 5 Syracuse Veterans Affairs Medical Center Syracuse, NY United States; 6 Center for Health Promotion and Disease Prevention University of North Carolina Chapel Hill, NC United States; 7 Department of Maternal and Child Health University of North Carolina Chapel Hill, NC United States; 8 Division of General Medicine and Clinical Epidemiology, Department of Medicine University of North Carolina School of Medicine Chapel Hill, NC United States; 9 Public Health Sciences Division Fred Hutchinson Cancer Center Seattle, WA United States

**Keywords:** community pharmacy, telehealth, telemedicine, telepharmacy, pharmacy service quality, patient willingness, willingness, cross-sectional, national survey, telehealth cost

## Abstract

**Background:**

Telehealth (telemedicine and telepharmacy) services increase access to patient services and ensure continuity of care. However, few studies have assessed factors that influence patients’ willingness to use telehealth services, and we sought to investigate this.

**Objective:**

This study aims to examine respondents’ (aged between 45 and 75 years) willingness to use telehealth services (telepharmacy and telemedicine) and the correlates of the willingness to use telehealth services.

**Methods:**

We administered a cross-sectional national survey of 1045 noninstitutionalized US adults aged between 45 and 75 years in March and April 2021. Multiple logistic regression analyses were used to identify demographic and health service use correlates of self-reported willingness to use telehealth services.

**Results:**

Overall willingness to use telemedicine was high (674/1045, 64.5%). Adults aged 55 years and older were less willing to use telemedicine (aged between 55 and 64 years: odds ratio [OR] 0.61, 95% CI 0.42-0.86; aged 65 years or older: OR 0.33, 95% CI 0.22-0.49) than those younger than 55 years. Those with a regular provider (OR 1.01, 95% CI 1-1.02) and long travel times (OR 1.75, 95% CI 1.03-2.98) were more willing to use telemedicine compared to those without a regular provider and had shorter travel times, respectively. Willingness to use telemedicine services increased from 64.5% (674/1045) to 83% (867/1045) if the service was low-cost or insurance-covered, was with their existing health care provider, or was easy-to-use. Overall willingness to use telepharmacy was 76.7% (801/1045). Adults aged older than 55 years were less willing to use telepharmacy (aged between 55 and 64 years: OR 0.57, 95% CI 0.38-0.86; aged 65 years or older: OR 0.24, 95% CI 0.15-0.37) than those younger than 55 years. Those who rated pharmacy service quality higher were more willing to use telepharmacy (OR 1.06, 95% CI 1.03-1.09) than those who did not.

**Conclusions:**

Respondents were generally willing to use telehealth (telemedicine and telepharmacy) services, but the likelihood of their being willing to use telehealth decreased as they were older. For those initially unwilling (aged 55 years or older) to use telemedicine services, inexpensive or insurance-covered services were acceptable.

## Introduction

Telehealth (eg, telemedicine and telepharmacy) has increased access to and sustainment of inpatient and outpatient services, specialty services, and pharmacy dispensing and nondispensing services (eg, vaccination counseling) [1,2]. Telehealth is defined as the provision of clinical care, patient education, and public health remotely through secure telecommunication technologies (eg, Zoom; Zoom Video Communications) [3], to enhance access to high-quality care [1,4-6]. Telehealth is a broad term that encompasses remote health care services provided by health care professionals, including physicians, nurses, and pharmacists [7,8]. Telepharmacy specifically refers to the delivery of pharmacy services, including remote dispensing, medication therapy management, and remote consultation [2,9,10]. Telemedicine is defined as the provision of clinic and diagnostic services remotely by physicians through telecommunication platforms [7]. Hence, telehealth includes telemedicine and telepharmacy.

Past studies have reported the success of remote clinic or doctor’s appointments remotely providing mental health services, medication dispensing, patient education, and counseling [5], which can improve medication adherence and outcomes in chronic disease management [6,8,11,12]. Precautions during the COVID-19 pandemic, such as social distancing, necessitated telehealth services in health care settings [10]. Through this shift, the perceived value of telehealth services has grown, as it means a lower risk of infection for people who are immunocompromised, lower travel expenses, convenience for patients, increased access to health services, and better continuity of care [13,14]. Additionally, the Coronavirus Aid Relief, Economic Security Act (CARES Act) of 2020 and the Centers for Medicare and Medicaid Services eased restrictions on Medicare reimbursement for telehealth services, further increasing access to health care services delivered through telehealth [15,16].

Telehealth services (including telemedicine and telepharmacy) show great promise to improve health care access equity, but few studies examine factors associated with patients’ willingness to use telehealth among older US adults. The Andersen health care utilization model posits that predisposing and enabling factors influence what type of care and how much care a person uses [17]. Predisposing factors for using telehealth include distance to the nearest physical provider or pharmacy, patient age, insurance status, or income. Telehealth (telepharmacy and telemedicine) could be considered enabling factors to increase access to patient care services. We aimed to evaluate the correlates of patients’ willingness to use telehealth (referred to as telemedicine and telepharmacy in this study) services in clinical and pharmacy settings among US adults aged between 45 and 75 years.

## Methods

### Participants and Procedures

We conducted a cross-sectional survey, the National PharmFIT Patient Survey, on noninstitutionalized US adults aged between 45 and 75 years who were eligible for colorectal cancer screening. More details about our participants and survey sampling procedures are published elsewhere [18] and briefly described here. Our national survey included members of market research panels managed by Qualtrics, a web-based survey research company [19]. We applied sampling quotas for participants to match the 2010 US census data for race, ethnicity, and sex and oversampled rural residents to comprise a third of our sample. Respondents provided informed consent and completed the survey between March and April 2021. This study sample includes 1045 panelists who were eligible and completed the survey. Using the American Association for Public Opinion Research response rate 4 [20], we accounted for ineligibility, data quality issues, and overquotas, yielding a response rate of 62% (1274/2054). Respondents represented all 50 states; Washington, DC; and Puerto Rico.

### Measures

#### Overview

The National PharmFIT Patient Survey developed by our multidisciplinary research team comprised 9 different domains: colorectal cancer screening experience, health care use, pharmacy type, PharmFIT program design, diffusion of innovation, willingness to use PharmFIT, follow-up care, telehealth (telemedicine and telepharmacy), and demographics. In this study, we evaluated telehealth items embedded in the National PharmFIT Patient Survey [18].

#### Telemedicine and Telepharmacy

In this study, we define telemedicine and telepharmacy as the delivery of health services remotely by a health care provider or pharmacist [7,8], respectively. Hereinafter, we refer to telehealth as both telemedicine and telepharmacy. A total of 3 survey items assessed respondents’ previous use of and willingness to use telehealth services. The first item assessed respondents’ willingness to use telemedicine services with a health care provider. The item used a 5-point response scale ranging from “strongly disagree” (1) to “strongly agree” (5). Those who strongly disagreed, disagreed, or neither agreed nor disagreed were categorized as “not willing to use telemedicine” (coded 0), while those who agreed or strongly agreed were categorized as “willing to use telemedicine” (coded 1). Participants who were “not willing to use telemedicine” received a follow-up multiple-choice question (second item) assessing under which conditions they would be willing to use telemedicine services with a health care provider. These conditions included: (1) getting an appointment sooner than an in-person appointment, (2) getting an appointment with the respondent’s usual health care provider, (3) whether the system was easy to use, (4) whether the system was private, (5) whether the appointments were available during extended hours, (6) whether insurance covered visits, (7) other reasons, or (8) none of these. The third item was a multiple-choice question assessing which health care services the participant would be willing to access through telepharmacy. We recoded this item as a dichotomous variable: 0 for “not willing to use telepharmacy” (for those who selected “none of these services” or gave a negative response in the open-ended response option) or 1 for “willing to use telepharmacy” (for those who selected any pharmacy service or stated other pharmacy services in the open-ended response section).

#### Demographic and Health Use Characteristics

The survey assessed respondents’ sociodemographic characteristics, their usual place of receiving care, whether they had a regular provider, travel time to their regular provider, the type of pharmacy they typically use for health care needs, travel time to their typical pharmacy, and their perceptions of pharmacy service quality. Demographic characteristics included age (categorized in 10-year increments), gender, race, ethnicity, insurance type, educational attainment, household income (US $20,000 increments), and rurality. Rurality was grouped into “rural” and “nonrural” by mapping respondents’ zip codes to rural-urban continuum codes [21]. Pharmacy service quality rating was developed as a composite score from items assessing how sympathetic, trustworthy, and responsive the pharmacy team was and how safe and familiar respondents felt with the pharmacy staff. These items used a 5-point response scale ranging from “strongly disagree” (1) to “strongly agree” (5) and were summarized as a continuous score, ranging from 0 to 30. High scores indicated better pharmacy service quality. The full survey instrument can be accessed online [22].

#### Statistical Analyses

We performed summary statistics to describe the demographic and health use factors (age, gender, race, ethnicity, insurance type, rurality, education, income, health status, place of care, regular source of care, pharmacy service quality rating, and pharmacy type) of our sample. Next, using willingness to use telemedicine and telepharmacy as our outcomes and the demographic and health usage factors listed above, which we determined a priori from Andersen’s model and past literature as the predictors [17,23-25], we conducted multiple logistic regressions to assess the correlates of respondents’ willingness to use telemedicine and telepharmacy. Finally, we described different conditions under which respondents indicated willingness to use telemedicine if they were initially unwilling. All analyses used a critical α=.05 and were conducted using Stata (version 16.1; StataCorp).

### Ethical Considerations

The University of North Carolina institutional review board approved this study (18-1337). All participants provided informed consent online before accessing and completing the survey.

## Results

### Respondents’ Characteristics and Willingness to Use Telemedicine and Telepharmacy Services

The majority of respondents were older than 55 years, non-Hispanic White, nonrural, had private insurance, and had household incomes below US $80,000. At least two-thirds of respondents were willing to use telemedicine (674/1045, 64.5%), and telepharmacy (801/1045, 76.7%; Table 1).

**Table 1:** Characteristics of respondents (N=1045).

Characteristics										Value		
**Sex, n (%)**												
	Male								523 (50)			
	Female								522 (50)			
**Age (years), n (%)**												
			45-54						348 (33)			
			55-64						348 (33)			
			≥65						349 (33)			
**Race, n (%)**												
				White					770 (74)			
				Black or African American					134 (13)			
				Asian					68 (7)			
				Multiracial or other					73 (7)			
**Ethnicity, n (%)**												
		Not Hispanic or Latinx							919 (88)			
		Hispanic or Latinx							126 (12)			
**Insurance type, n (%)**												
							Private		569 (56)			
							Public		384 (38)			
							Uninsured		60 (6)			
**Rurality, n (%)**												
							Not rural		731 (70)			
							Rural		314 (31)			
**Education, n (%)**												
				High school or less					217 (21)			
				Some college					408 (39)			
				College degree					259 (25)			
				Graduate education or higher					161 (15)			
**Household income (US $), n (%)**												
							<20,000		156 (15)			
							20,000-39,000		247 (24)			
							40,000-59,000		219 (21)			
							60,000-79,000		151 (14)			
							80,000-99,000		89 (9)			
							>100,000		183 (18)			
**Health status, n (%)**												
					Excellent					77 (7)		
					Very good					301 (29)		
					Good					443 (42)		
							Fair		190 (18)			
							Poor		34 (3)			
**Place to receive health care, n (%)**												
								A doctor’s office or health care center	895 (85)			
								Urgent care center or clinic in a drug store or grocery store	73 (7)			
								Emergency room	32 (3)			
								A VA^a^ medical center or VA outpatient clinic	39 (4)			
								Some other place	6 (1)			
**Have provider as a regular source of care, n (%)**												
					No					112 (10)		
					Yes					933 (89)		
**Willingness to use telemedicine, n (%)**												
					No					371 (36)		
					Yes					674 (64)		
Travel time to provider (minutes), mean (SD)										18.73 (14.19)		
**Pharmacy type, n (%)**												
						A retail chain pharmacy (like CVS^b^ or Walgreens)			515 (49)			
						A pharmacy in a grocery store (like Kroger or Albertsons or Safeway)			164 (16)			
						A pharmacy in a department store or wholesaler (like Walmart or Costco)			166 (16)			
						A pharmacy in a clinic or hospital where medical care is received			90 (9)			
						A local, independently owned pharmacy			110 (11)			
**Willingness to use telepharmacy, n (%)**												
						No			244 (23)			
						Yes			801 (77)			
Travel time to nearest pharmacy (minutes), mean (SD)										18.79 (14.51)		
Pharmacy service quality rating, mean (SD)										17.51 (4.91)		

^a^VA: Veteran’s Affairs.

^b^CVS: A retail pharmacy chain in the United States.

### Correlates of Respondents’ Willingness to Use Telemedicine Services

In the multiple logistic regression model, compared to participants aged between 45 and 54 years, respondents aged between 55 and 64 years (odds ratio [OR] 0.61, 95% CI 0.42-0.86) and respondents aged 65 years or older (OR 0.33, 95% CI 0.22-0.49) were less likely to be willing to use telemedicine. Respondents who had a regular provider (OR 1.01, 95% CI 1-1.02) and had long travel times to their provider (OR 1.75, 95% CI 1.03-2.98) were more likely to be willing to use telemedicine (Table 2).

**Table 2:** Correlates of respondents’ willingness to use telehealth services (N=1045).

Characteristics											Willing to use telemedicine							Willing to use telepharmacy				
											Value		OR^a^ (95% CI)		*P* value		Value			OR (95% CI)		*P* value
**Sex, n/N (%)**																						
	Male										333/523 (63.7)		Reference		—^b^		400/523 (76.5)			Reference		—
	Female										341/522 (65.3)		1.12 (0.84-1.48)		.44		401/522 (76.8)			1.10 (0.80-1.5)		.57
**Age (years), n/N (%)**																						
		45-54								260/348 (74.7)		Reference		—		296/348 (85.1)			Reference		—	
		55-64								231/348 (66.4)		0.61 (0.42-0.86)		.01		275/348 (79)			0.57 (0.38-0.86)		.01	
		≥65								183/349 (52.4)		0.33 (0.22-0.49)		<.001		230/349 (65.9)			0.24 (0.15-0.37)		<.001	
**Race, n/N (%)**																						
						White				484/770 (62.9)		Reference		—		580/770 (75.3)			Reference		—	
						Black or African American				98/134 (73.1)		1.51 (0.97-2.37)		.07		109/134 (81.3)			1.37 (0.82-2.29)		.23	
						Asian				44/68 (65)		0.87 (0.49-1.54)		.64		53/68 (78)			1.17 (0.61-2.25)		.63	
						Multiracial or other				48/73 (66)		1.04 (0.59- 1.82)		.88		59/73 (81)			1.43 (0.73-2.79)		.30	
**Ethnicity, n/N (%)**																						
				Not Hispanic or Latinx						589/919 (64.1)		Reference		—		701/919 (76.3)			Reference		—	
				Hispanic or Latinx						85/126 (67.5)		1.02 (0.65-1.59)		.94		100/126 (79.4)			1.13 (0.67-1.90)		.64	
**Insurance type, n/N (%)**																						
								Private			272/384 (70.8)		0.63 (0.32-1.27)		.20		306/384 (79.7)			0.81 (0.39-1.67)		.57
								Public			338/569 (59.4)		0.61 (0.31-1.22)		.16		421/569 (74)			1.14 (0.56-2.33)		.71
								Uninsured			43/60 (72)		Reference		—		47/60 (78)			Reference		—
**Rurality, n/N (%)**																						
									Not rural	488/731 (66.8)		Reference		—		570/731 (78)			Reference		—	
									Rural	186/314 (59.2)		0.80 (0.58-1.1)		.17		231/314 (73.6)			0.75 (0.52-1.08)		.12	
**Education, n/N (%)**																						
									High school or less	129/217 (59.4)		Reference		—		161/217 (74.2)			Reference		—	
									Some college	255/408 (62.5)		1.02 (0.71-1.48)		.90		304/408 (74.5)			0.89 (0.59-1.35)		.60	
									College degree	180/259 (69.5)		1.37 (0.89-2.12)		.15		211/259 (81.5)			1.35 (0.83-2.22)		.23	
									Graduate education or higher	110/161 (68.3)		1.26 (0.76-2.08)		.37		125/161 (77.6)			1.16 (0.66-2.02)		.61	
**Household income (US $), n/N (%)**																						
					<20,000					97/156 (62.2)		Reference		—		117/156 (75)			Reference		—	
					20,000-39,000					156/247 (63.2)		1.11 (0.71-1.73)		.65		181/247 (73.3)			1.08 (0.65-1.78)		.77	
					40,000-59,000					123/219 (56.2)		0.86 (0.53-1.38)		.53		167/219 (76.3)			1.52 (0.88-2.64)		.13	
					60,000-79,000					109/151 (72.2)		1.59 (0.93-2.72)		.09		120/151 (79.5)			1.42 (0.77-2.6)		.26	
					80,000-99,000					59/89 (66)		1.07 (0.57-1.99)		.83		72/89 (81)			1.63 (0.78-3.39)		.19	
					>100,000					130/183 (71)		1.34 (0.78-2.32)		.29		144/183 (78.7)			1.34 (0.72-2.48)		.35	
**Health status, n/N (%)**																						
	Excellent										57/77 (74)		1.70 (0.68-4.26)		.26		63/77 (82)			0.85 (0.28-2.59)		.77
	Very good										189/301 (62.8)		1.09 (0.50-2.37)		.83		232/301 (77.1)			0.79 (0.30-2.1)		.63
	Good										287/443 (64.8)		1.28 (0.60-2.75)		.52		333/443 (75.2)			0.72 (0.28-1.88)		.50
	Fair										121/190 (63.7)		1.18 (0.54-2.62)		.68		145/190 (76.3)			0.74 (0.27-2)		.55
	Poor										20/34 (59)		Reference		—		28/34 (82)			Reference		—
**Place to receive health care^c^, n/N (%)**																						
			A doctor’s office or health care center							578/895 (64.6)		1.54 (0.66-3.58)		.32		—			—		—	
			Urgent care center or clinic in a drug store or grocery store							49/73 (67)		1.79 (0.71-5.53)		.22		—			—		—	
			Emergency room							18/32 (56)		Reference				—			—		—	
			A VA^d^ medical center or VA outpatient clinic							26/39 (67)		1.81 (0.61-5.35)		.29		—			—		—	
			Some other place							3/6 (50)		1.38 (0.18-10.75)		.76		—			—		—	
**Have provider as a regular source of care^c^, n/N (%)**																						
					No					65/112 (58)		Reference				—			—		—	
					Yes					609/933 (65)		1.75 (1.03-2.98)		.04		—			—		—	
Travel time to provider^c^, mean (SD)											18.73 (14.19)		1.01 (1-1.02)		.05		—			—		—
**Pharmacy type^e^, n/N (%)**																						
							A retail chain pharmacy (like CVS^f^ or Walgreens)			—		—		—		395/515 (76.7)			Reference		—	
							A pharmacy in a grocery store (like Kroger or Albertsons or Safeway)			—		—		—		125/164 (76.2)			0.99 (0.64-1.53)		.95	
							A pharmacy in a department store or wholesaler (like Walmart or Costco)			—		—		—		124/166 (74.7)			1.03 (0.67-1.60)		.89	
							A pharmacy in a clinic or hospital where you receive medical care			—		—		—		75/90 (83)			1.87 (0.98-3.54)		.06	
							A local, independently owned pharmacy			—		—		—		82/110 (74.5)			0.98 (0.58-1.66)		.94	
Travel time to nearest pharmacy^e^, mean (SD)											—		—				18.79 (14.51)			1 (0.99-1.01)		.73
Pharmacy service quality rating^e^, mean (SD)											—		—				17.51 (4.91)			1.06 (1.03-1.09)		<.001

^a^OR: odds ratio.

^b^Not available.

^c^Items specific to telemedicine.

^d^VA: Veteran’s Affairs.

^e^Items specific to telepharmacy.

^f^A retail pharmacy chain in the United States.

### Conditions That Make Respondents Willing to Use Telemedicine

A minority of respondents reported ambivalence toward or disagreed with the use of telemedicine (371/1045, 35.5%). About a third of these respondents indicated they would be willing to use telemedicine if appointments were low-cost or insurance-covered (135/371, 36.4%) or if the appointment was with their usual health care provider (125/371, 33.7%). About a quarter of these respondents were willing to use telemedicine if the platform was easy to use (98/371, 26.4%). To a lesser extent, respondents indicated the other conditions as important criteria to meet before they were willing to use telemedicine (Table 3). After accounting for the top 3 conditions for using telemedicine (low-cost or insurance-covered appointments, having the appointment with a health care provider, and a user-friendly platform), respondents’ willingness to use telemedicine increased from 64.5% (674/1045) to 82.9% (867/1045).

**Table 3:** Conditional use of telemedicine services with a health care provider (n=371).

“I would be willing to use telemedicine to communicate with a health care provider about my health only if...”	Respondents, n (%)
My insurance would pay for it, or it would be low-cost for me	135 (37)
My appointment was with my health care provider	125 (34)
The system was easy to use	98 (26)
The system was private	91 (25)
I could get an appointment sooner than an in-person appointment	66 (18)
The appointments were available during extended hours, such as evening	48 (13)

### Willingness to Use Patient Care Services Through Telepharmacy

Respondents were willing to use telepharmacy services for medication counseling (727/1045, 69.6%), vaccination consultation (511/1045, 48.9%), urgent care consultation (293/1045, 28%), chronic disease management (283/1045, 27.1%), travel medicine consultation (252/1045, 24.1%), genetic testing counseling (159/1045, 15.2%), and smoking cessation counseling (85/1045, 8.1%; Table 4).

**Table 4:** Proportion of respondents willing to use telepharmacy services (N=1045).

Patient care services	Respondents, n (%)
Medication counseling	727 (70)
Vaccination consultation	511 (49)
Urgent care consultation	252 (28)
Chronic disease management and education	283 (27)
Travel medicine consultation	252 (24)
Genetic testing counseling	159 (15)
Smoking or tobacco cessation counseling	85 (8)
Other	15 (1)

### Correlates of Respondents’ Willingness to Use Telepharmacy Services

In the multiple logistic regression model, compared to participants aged between 45 and 54 years, respondents aged between 55 and 64 years, (OR 0.57, 95% CI 0.38-0.86) and respondents aged 65 years or older (OR 0.24, 95% CI 0.15-0.37) were less likely to be willing to use telepharmacy services. Respondents who reported a higher pharmacy service quality rating were also more likely to be willing to use telepharmacy (OR 1.06, 95% CI 1.03-1.09; Table 2).

## Discussion

### Principal Results and Comparison With Previous Work

The capacity of telehealth (ie, telemedicine and telepharmacy) to expand access to health services, especially in remote areas and to marginalized populations, has been established [1,4,5]. This study adds to this literature by revealing that a substantial proportion of our national sample of older adults were willing to use telehealth (which refers to both telemedicine and telepharmacy in this study) services. Further, we found that age, having a regular provider, longer travel time to the provider, and perceived higher pharmacy service quality were factors associated with willingness to use telehealth services.

We found that older adults (aged 55 years and older) were less willing to access telehealth services than those aged between 45 and 54 years. Previous studies have reported similar results and noted that older patients could be more hesitant to use telehealth services in general due to their inexperience with modern technology and the perception that their health care needs cannot be effectively addressed in remote appointments [24-26]. Another study that assessed the readiness of adults to use telehealth reported that among people aged 65 years or older in the US population, over 30% were not ready to access medical services through telehealth, with 20% of this group being resistant due to physical disabilities such as difficulty seeing and hearing [24]. As such, to increase the use of telehealth, health care settings should develop educational materials or training, as well as more user-friendly telehealth models for diverse adult populations that account for disabilities like visual and hearing impairment [27-29].

We also found that participants who had a regular health care provider and reported higher appraisals of pharmacy service quality were more willing to use telemedicine and telepharmacy, respectively. Strong rapport in the form of familiarity, trustworthiness, and responsiveness are established characteristics of good patient-provider relationships [30,31]. While patients tend to have established care with primary care and other specialty providers, they may be less likely to have an “established” pharmacy and, by extension, usual pharmacists [32,33],​ from whom they receive care. Our findings suggest that the use of telepharmacy services among older adults may increase if community pharmacists are able to establish better interpersonal connections with their patients. This could be achieved by assuming a patient-centered approach in the delivery of pharmacy services or constant patient engagement through regular check-ins to address patients’ health-related questions.

Unsurprisingly, we found that increased travel time to a health care provider was associated with a higher willingness to use telemedicine. Barriers to attending in-person clinic appointments are well established, such as access to reliable transportation, taking time off work, or childcare services. Similar to our finding, a systematic review showed that patients were more satisfied with a telemedicine option because of lower financial costs and resource commitments [34]. Multiple studies have shown that with telemedicine, patients were able to save hours on the road, lower transportation expenses, shorten clinic wait times, and avoid missing work [14,35,36]. These external factors can be nontrivial, especially for older adults on fixed incomes or who have difficulty traveling and should be highlighted as benefits of telehealth services in both medical and pharmacy settings.

Past studies have demonstrated that low uptake of telehealth may be due to unfamiliarity with the telehealth technology or the cost of the telehealth service [24-26].​ This study revealed similar reasons why people may not want to participate in telemedicine, such as high costs or no insurance coverage, not having an appointment with their provider, and complicated telemedicine platforms. As clinics and pharmacies are adopting telehealth services, they should consider the characteristics of their patient populations and select simpler and more user-friendly platforms. From a policy standpoint, accounting for the rollback of compensation for telehealth services by the federal government, insurance companies and regulators should consider ways to create and maintain reimbursement models and incentives that could increase the use of telehealth services, as these services may help contain costs and be more patient-centered and convenient to some [37,38].

### Strengths and Limitations

Strengths of this study include oversampling of medically underserved groups, as evidenced by our sample having a higher proportion of rural residents and those with low socioeconomic status. Although a convenience sample, our quotas matched the US census. Another strength is that we evaluated the correlates of willingness to use telemedicine and telepharmacy. Previous studies have either focused on telemedicine or telepharmacy only and may not have evaluated factors that lead to telemedicine service use. Additionally, our survey was administered during the pandemic, so familiarity with and use of telehealth likely increased during the time of this study, increasing the timeliness and relevance of our results.

A limitation to consider is that this study was cross-sectional, so we are unable to establish temporality and whether the uptake of telehealth services among this population increased over time. Also, this was an internet-based survey, and all participants had internet access, so we could not assess telehealth needs or preferences among those that may have poorer access to these technologies. Another limitation was that we sampled specific adults that met the criteria for our larger PharmFIT Patient Survey, which may not be wholly representative of the older adult population. Additional studies might need to be conducted to assess the overall generalizability of our findings to the older adult population. Also, we could not assess whether a participant’s health plan covered telehealth services. However, due to the COVID-19 pandemic, telehealth services have become more widespread, more types of patient care services are provided remotely, and insurance plans more readily cover the costs of these services [39].

### Conclusion

Our results revealed that respondents were generally willing to use telehealth (telemedicine and telepharmacy) services, but the likelihood of them being willing to use these services decreased as respondents were older. We also found that for those unwilling to use telemedicine services, if certain stipulations (eg, inexpensive services) were met, then their willingness to use these services increased. Telehealth services have increased during the COVID-19 pandemic, showing the relevance of this approach for health care providers to adopt across the country in closing the gap in access to health services [10]. There were uncertainties about whether telehealth services would continue to be reimbursed after the pandemic, but evidence suggests that federal and private insurers would continue to reimburse these services [40,41]. It is critical that telehealth services continue to be available, as they improve equitable access and have a tendency to reduce health-related costs [34]. Hence, to increase adoption, more considerations, such as providing low-cost or easy-to-use telehealth options, are needed to design telehealth platforms that can accommodate patients with certain characteristics (eg, older adults). Similarly, providers in clinics and pharmacies need to be aware of situations that increase the willingness to use telehealth services, such as establishing a good rapport with patients, and decrease the willingness, such as a lack of insurance coverage.

## Abbreviations

CARES Act: Coronavirus Aid Relief, Economic Security Act
OR: odds ratio

## Data Availability

The data used in this study are available on request from the corresponding author.

## References

[1] Kovačević M, Ćulafić M, Vezmar Kovačević S, Borjanić S, Keleč B, Miljković B, Amidžić R (2022). Telepharmacy service experience during the COVID-19 pandemic in the Republic of Srpska, Bosnia and Herzegovina. Health Soc Care Community.

[2] Le T, Toscani M, Colaizzi J (2020). Telepharmacy: a new paradigm for our profession. J Pharm Pract.

[3] (2024). Office for the advancement of telehealth. Health Resources & Services Administration.

[4] Pathak S, Haynes M, Qato DM, Urick BY (2020). Telepharmacy and quality of medication use in rural areas, 2013-2019. Prev Chronic Dis.

[5] Inch J, Notman F, Watson M, Green D, Baird R, Ferguson J, Hind C, McKinstry B, Strath A, Bond C, Telepharmacy Research Team (2017). Tele-pharmacy in rural Scotland: a proof of concept study. Int J Pharm Pract.

[6] Baldoni S, Amenta F, Ricci G (2019). Telepharmacy services: present status and future perspectives: a review. Medicina (Kaunas).

[7] (2024). Telehealth, telemedicine, and telecare: what's what?. Federal Communications Commission.

[8] Perisetti A, Goyal H (2021). Successful distancing: telemedicine in gastroenterology and hepatology during the COVID-19 pandemic. Dig Dis Sci.

[9] (2021). Regulation of telepharmacy practice. Federal Register.

[10] Unni EJ, Patel K, Beazer IR, Hung M (2021). Telepharmacy during COVID-19: a scoping review. Pharmacy (Basel).

[11] Maxwell LG, McFarland MS, Baker JW, Cassidy RF (2016). Evaluation of the impact of a pharmacist-Led telehealth clinic on diabetes-related goals of therapy in a veteran population. Pharmacotherapy.

[12] Varker T, Brand RM, Ward J, Terhaag S, Phelps A (2019). Efficacy of synchronous telepsychology interventions for people with anxiety, depression, posttraumatic stress disorder, and adjustment disorder: a rapid evidence assessment. Psychol Serv.

[13] Ramsey A, Yang L, Vadamalai K, Mustafa SS (2020). Appointment characteristics in an allergy/immunology practice in the immediate aftermath of COVID-19 restrictions. J Allergy Clin Immunol Pract.

[14] Nguyen M, Waller M, Pandya A, Portnoy J (2020). A review of patient and provider satisfaction with telemedicine. Curr Allergy Asthma Rep.

[15] Cubanski J, Kates J, Tolbert J, Guth M, Pollitz K, Freed M (2023). What happens when COVID-19 emergency declarations end? Implications for coverage, costs, and access. KFF.

[16] (2023). CARES act: AMA COVID-19 pandemic telehealth fact sheet. American Medical Association.

[17] Andersen RM (1995). Revisiting the behavioral model and access to medical care: does it matter?. J Health Soc Behav.

[18] Brenner AT, Waters AR, Wangen M, Rohweder C, Odebunmi O, Marciniak MW, Ferrari RM, Wheeler SB, Shah PD (2023). Patient preferences for the design of a pharmacy-based colorectal cancer screening program. Cancer Causes Control.

[19] (2023). The leading experience management software. Qualtrics XM.

[20] (2016). Standard definitions: final dispositions of case codes and outcome rates for surveys. 9th Edition. The American Association for Public Opinion Research.

[21] (2023). Rural-Urban Continuum Codes. U.S. Department of Agriculture: Economic Research Service.

[22] (2023). PharmFIT - the 4CNC Core Project (2019-2024). Dataverse.

[23] Mackwood MB, Tosteson TD, Alford-Teaster JA, Curtis KM, Lowry ML, Snide JA, Zhao W, Tosteson ANA (2022). Factors influencing telemedicine use at a northern new England cancer center during the COVID-19 pandemic. JCO Oncol Pract.

[24] Lam K, Lu AD, Shi Y, Covinsky KE (2020). Assessing telemedicine unreadiness among older adults in the United States during the COVID-19 pandemic. JAMA Intern Med.

[25] Kong SS, Otalora Rojas Lilian A, Ashour A, Robinson M, Hosterman T, Bhanusali N (2021). Ability and willingness to utilize telemedicine among rheumatology patients-a cross-sectional survey. Clin Rheumatol.

[26] Omboni S, Tenti M (2019). Telepharmacy for the management of cardiovascular patients in the community. Trends Cardiovasc Med.

[27] (2023). Improving access to telehealth. Telehealth.HHS.gov.

[28] (2024). Telehealth. ADA.gov: U.S. Department of Justice Civil Rights Division.

[29] Valdez RS, Rogers CC, Claypool H, Trieshmann L, Frye O, Wellbeloved-Stone C, Kushalnagar P (2021). Ensuring full participation of people with disabilities in an era of telehealth. J Am Med Inform Assoc.

[30] Worley MM (2006). Testing a pharmacist-patient relationship quality model among older persons with diabetes. Res Social Adm Pharm.

[31] Esmalipour R, Salary P, Shojaei A (2021). Trust-building in the pharmacist-patient relationship: a qualitative study. Iran J Pharm Res.

[32] Mc Namara KP, Dunbar JA, Philpot B, Marriott JL, Reddy P, Janus ED (2012). Potential of pharmacists to help reduce the burden of poorly managed cardiovascular risk. Aust J Rural Health.

[33] Smith KB, Humphreys JS, Lenard Y, Jones JA, Prince V, Han GS (2004). Still the doctor - by a country mile! Preferences for health services in two country towns in north-west New South Wales. Med J Aust.

[34] Kruse CS, Krowski N, Rodriguez B, Tran L, Vela J, Brooks M (2017). Telehealth and patient satisfaction: A systematic review and narrative analysis. BMJ Open.

[35] Mustafa SS, Yang L, Mortezavi M, Vadamalai K, Ramsey A (2020). Patient satisfaction with telemedicine encounters in an allergy and immunology practice during the coronavirus disease 2019 pandemic. Ann Allergy Asthma Immunol.

[36] Taylor L, Capling H, Portnoy JM (2018). Administering a telemedicine program. Curr Allergy Asthma Rep.

[37] Niu B, Mukhtarova N, Alagoz O, Hoppe K (2022). Cost-effectiveness of telehealth with remote patient monitoring for postpartum hypertension. J Matern Fetal Neonatal Med.

[38] Agha Z, Schapira RM, Maker AH (2002). Cost effectiveness of telemedicine for the delivery of outpatient pulmonary care to a rural population. Telemed J E Health.

[39] Demeke HB, Pao LZ, Clark H, Romero L, Neri A, Shah R, McDow KB, Tindall E, Iqbal NJ, Hatfield-Timajchy K, Bolton J, Le X, Hair B, Campbell S, Bui C, Sandhu P, Nwaise I, Armstrong PA, Rose MA (2020). Telehealth practice among health centers during the COVID-19 pandemic - United States, July 11-17, 2020. MMWR Morb Mortal Wkly Rep.

[40] Salmanizadeh F, Ameri A, Bahaadinbeigy K (2022). Methods of reimbursement for telemedicine services: a scoping review. Med J Islam Repub Iran.

[41] (2024). MLN90175 - Telehealth Services. Centers for Medicare and Medicaid Services. Medicare Learning Network.

